# A network-based approach to classify the three domains of life

**DOI:** 10.1186/1745-6150-6-53

**Published:** 2011-10-13

**Authors:** Laurin AJ Mueller, Karl G Kugler, Michael Netzer, Armin Graber, Matthias Dehmer

**Affiliations:** 1 Institute for Bioinformatics and Translational Research, Department of Biomedical Sciences and Engineering, University for Health Sciences, Medical Informatics and Technology (UMIT), Austria; 2 Institute of Electrical, Electronic and Bioengineering, Department of Biomedical Sciences and Engineering, University for Health Sciences, Medical Informatics and Technology (UMIT), Austria

## Abstract

**Background:**

Identifying group-specific characteristics in metabolic networks can provide better insight into evolutionary developments. Here, we present an approach to classify the three domains of life using topological information about the underlying metabolic networks. These networks have been shown to share domain-independent structural similarities, which pose a special challenge for our endeavour. We quantify specific structural information by using topological network descriptors to classify this set of metabolic networks. Such measures quantify the structural complexity of the underlying networks. In this study, we use such measures to capture domain-specific structural features of the metabolic networks to classify the data set. So far, it has been a challenging undertaking to examine what kind of structural complexity such measures do detect. In this paper, we apply two groups of topological network descriptors to metabolic networks and evaluate their classification performance. Moreover, we combine the two groups to perform a feature selection to estimate the structural features with the highest classification ability in order to optimize the classification performance.

**Results:**

By combining the two groups, we can identify seven topological network descriptors that show a group-specific characteristic by ANOVA. A multivariate analysis using feature selection and supervised machine learning leads to a reasonable classification performance with a weighted *F*-score of 83.7% and an accuracy of 83.9%. We further demonstrate that our approach outperforms alternative methods. Also, our results reveal that entropy-based descriptors show the highest classification ability for this set of networks.

**Conclusions:**

Our results show that these particular topological network descriptors are able to capture domain-specific structural characteristics for classifying metabolic networks between the three domains of life.

## Background

The interlinkage of enzymes into metabolic reactions allows catabolic and anabolic processes to provide organisms with energy and the building blocks of cell functions [1]. These interactions can be represented as metabolic networks [2]. The analysis of such networks gives insight into the functions of various processes, and is essential for understanding basic biological questions [3]. Typical analyses comprise studying vertex degrees or the paths between vertices. For instance, Jeong et al. demonstrated that despite their evolutionary distances, the domains of life share significant similarities in the topology of their metabolic networks [2]. They pointed out that a set of 43 organisms, representing the three domains of life (Archaea, Bacteria, Eukaryote), appeared to be scale-free and follow a power law distribution [2]. Additionally, they found network diameters to be relatively constant despite network size.

By performing a large-scale structural analysis of metabolic networks, it has been reported that hierarchical clusters of topological modules overlap with known metabolic functions [4]. In recent work, Ebenhöh and Handorf proposed strategies to characterize organisms with respect to their underlying metabolic networks. They introduced a measure for calculating the distance between organisms based on the carbon utilization spectra and the nutrient profiles of the metabolic networks [5]. To classify pathways through metabolic networks, Hancock and Mamitsuka use a Markov mixture model [6]. This model identifies pathways in metabolic networks in order to build a classifier. Zhu and Qin used basic network measures (e.g. clustering coefficient and average betweenness) and network motifs for a structural comparison of 11 metabolic networks [7]. The approach introduced in the present paper determines structural features by utilizing topological network descriptors, in order to classify metabolic networks of 43 organisms.

To analyse networks structurally, various topological network descriptors have been developed [8]. Such descriptors capture different structural features of networks and have proved to be useful in characterizing molecular networks [8-10]. In particular, it has been demonstrated that information-theoretic measures [8], interpreted as the entropy of the underlying graph topology, capture significant structural information [10-13]. Additionally, Dehmer et al. developed novel descriptors to analyse biological networks [14]. Hence, we hypothesize that these measures can be successfully applied to to capture topological properties of metabolic networks for classifying them with a reasonable classification performance. To calculate the topological network descriptors we used the R-package QuACN[15].

In general, graph classification is a challenging problem and has been tackled by using different methods [16-18] from exact and inexact graph matching [16,17]. Goh et al. use the betweenness centrality to classify different types of scale-free networks into two classes [19]. In more biologically motivated related work performed by Li et al. [20], graph kernels for machine learning were used to predict gene functions. Chuang et al. [21] used subnetworks to train a classifier for the detection of breast cancer metastasis.

**Figure 1:**
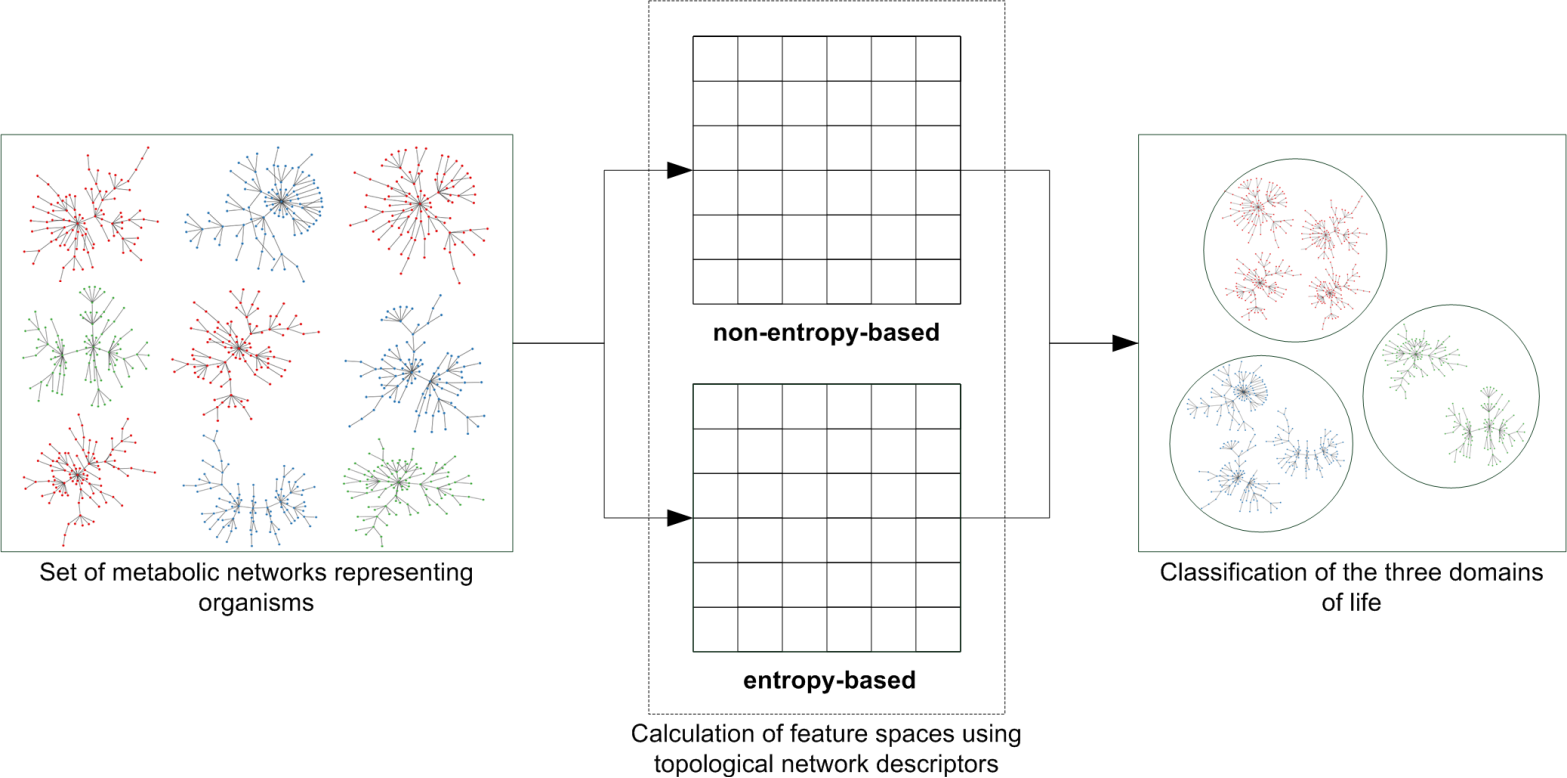
**Illustration of the main contribution of this paper**. This figure illustrates the main contribution of this paper. Our approach uses two groups of topological network descriptors (non entropy-based and entropy-based) to estimate their performance in classifying metabolic networks. Moreover, we combine the groups and apply feature selection.

The major contribution of the present paper, as illustrated in Figure 1, is the study of topological network descriptors and the evaluation of their performance when classifying the domains of life as presented in the data by Jeong et al. [2]. Therefore, we use different groups of topological network measures as input for supervised machine learning techniques in order to comprehensively capture topological network properties for classification. To our best knowledge, such an approach has not yet been employed on metabolic networks.

We introduce this approach for several reasons. Note that exact graph matching such as the Zelinka distance [22,23] is not applicable to calculate the distance between the networks. The size of the networks makes it unfeasible for us to pursue this endeavour, as the complexity of calculating the Zelinka distance between two networks is NP-complete [24]. Moreover, we would have to compare the networks with each other, leading to $\frac{n\left(n-1\right)}{2}$ comparisons.

Therefore, we apply a network-descriptor based approach. The calculation of the descriptors requires polynomial time complexity. However, a single network descriptor might be insufficient for capturing the topology of a complex network. For this reason, our approach is based on the combination of different topological network descriptors, which are prioritized and selected using feature selection. Clearly, this information can be used for performing the classification.

This paper is structured as follows: *Material and Methods *provide an overview of the applied methods. The results of our study are explained in the *Results *section, followed by a summary and the discussion of the results. The section *Conclusions and Outlook *concludes the study and outlines future work.

## Materials and methods

As hypothesis, we claim to have achieved a reasonable classification of the three domains of life by using only structural features of their underlying metabolic networks. Therefore, we make use of 43 metabolic networks introduced by Jeong et al. [2]. They point out that these networks show basic, domain-independent structural similarities. This makes distinguishing between the three domains of life a challenging task. To tackle this problem, our aim is to find complex topological features that allow detecting domain-specific similarities.

Therefore, we calculate two groups of topological descriptors for each network and estimate their ability to discriminate between the three domains of life. Moreover, we combine the two groups to a set of 33 features, which we later use for ANOVA testing. We compare the classification performance of the two groups with all combined descriptors using feature selection and supervised machine learning. Finally, we compare this approach with an alternative statistical degree-based method.

### Data

For our analysis we use the metabolic network data of Jeong et al. [2], where the information on the underlying biochemical reactions is based on the WIT database [25]. The original data set comprises 43 organisms represented as directed networks. The vertices in these networks represent substrates that are connected by metabolic reactions. The 43 organisms can be divided into three classes, which represent the domains of life Archae (*n_A _*= 6), Bacterium (*n_B _*= 32), and Eukaryote (*n_E _*= 5). Note that this data set is highly imbalanced (skewed class distribution). As the implementation of QuACN[15] is only capable of handling undirected networks, we transform the original data into undirected networks. After constructing these networks, the largest connected component is extracted for our analysis, as connected graphs are required by most of the network measures utilized.

### Topological Network Descriptors

In order to perform a feasible classification, we exploit sophisticated network descriptors to capture domain-specific topological complexity in a meaningful way [8,26]. In particular, we use topological network descriptors to quantify domain-specific topological properties. Each descriptor calculates a numerical value that quantifies specific topological characteristics of the underlying network.

The interpretation of the structural properties and complexity of the applied measures in detail, is a challenging and still ongoing task [27].

The recently developed R package QuACN contains four different groups of topological network descriptors to analyse complex biological networks [15]:

1. *Descriptors based on distances in a graph: *This class contains measures [9] based on distances between nodes.

2. *Descriptors based on other graph invariants: *The descriptors in this class use graph invariants other than distances, e.g., degree, number of nodes, number of edges, etc.

3. *Partition-based graph entropy descriptors: *These measures use an arbitrary graph invariant and an equivalence criteria to induce partitions. A probability value is calculated for each partition to determine the entropy based on Shannon's entropy [28].

4. *Parametric graph entropy measures called Dehmer-entropy: *To determine the entropy, measures of this class [8,10], assign a probability value to each vertex of a graph, using so-called information functionals (IFs).

A detailed description of the employed descriptors would go beyond the scope of this manuscript. For a better understanding of the descriptors used, see the vignette of QuACN or the corresponding literature. For example, Todeschini et al. [29] lists a large selection of topological network descriptors and Dehmer and Mowshowitz discuss entropy-based descriptors in detail [8].

We use QuACN as it is, as far as we know, the only available software package that contains sophisticated measures such as the parametric graph entropy measures (Dehmer entropy). Calculating the 33 descriptors that are implemented in QuACN version 1.0 results in a data matrix containing 43 samples (metabolic networks) and 33 features (topological descriptors).

This matrix is used for further analysis. To estimate the classification ability of different groups of topological network descriptors, we combine groups 1 and 2 into a group of *non entropy-based *descriptors (NEBD). The other group, *entropy-based *descriptors (EBD), is formed by merging groups 3 and 4. Based on previous observations, we expect EBD to perform better on classifying this set of networks.

### Univariate Analysis

Initially we test the topological network descriptors for a domain-specific effect. Therefore, we apply one way ANOVA [30] tests on each of the 33 descriptors. We correct for multiple hypothesis testing by calculating the *q*-values (adjusted *p*-values) using the false discovery rate (FDR) [31]. We used the statistical programming language R [32] for this analysis.

### Feature Selection

Feature selection is an essential step before building predictive models from biomedical data [33]. Feature selection methods can be classified into filters, wrappers, and embedded methods [34]. Wrappers employ learning algorithms to evaluate the discriminatory ability of feature subsets using heuristic approaches to search the space of possible feature subsets. In general, feature subsets selected by wrappers are highly discriminative, but wrappers have high computational costs. On the contrary, the search for an optimal subset of features is built into the classifier of embedded methods: thus they are less computationally intensive than wrappers [34]. Filter methods select features based on their ability to discriminate two or more predefined classes. Filters are independent of a learning algorithm, efficient, and permit of prioritizing features, which is particularly important for biological interpretation purposes [35].

In our experimental setup we use three filter methods, which are again one way ANOVA, Information Gain (IG) [36], and ReliefF (RF) [37]. The IG feature selection method is based on an entropy measure [36]. RF is a multivariate correlation-based feature selection method that compares feature values of the *k *nearest instances for the same and the other classes [37].

Leave-One-Out Cross-Validation (LOOCV) is applied to validate the feature rankings by subdividing the dataset into *m *(*m *= the number of instances) partitions. The feature selection process is then repeated *m *times, using *m *- 1 partitions for generating the feature ranking. In our particular case, *m *= 43. Finally, *m *rankings for each partitioning are calculated, and the mean ± sd (standard deviation) score of the selected feature subset is calculated [35].

### Supervised Machine Learning

In our experimental setup we use three important classifiers, which are the *k*-nearest neighbour (*k*-nn), Naive Bayes, and logistic regression. *k*-nn is an instance-based learner, assigning a new instance to the majority class of the *k *nearest neighbours of the training set [38]. Naive Bayes is a probabilistic classifier based on Bayes' rule of conditional probability assuming class independence [39]. Logistic Regression Analysis [40] calculates the posterior probabilities of the classes using linear functions. A logit transformation ensures that the predicted probabilities range between 0 and 1. A general and simple way to address multiclass problems in logistic regression is known as pairwise classification, where a classifier is built for each pair of classes [38]. To estimate the performance and reduce the selection bias of the classifiers, we use external Leave-One-Out Cross-Validation (LOOCV) [41,42]. Therefore, we perform the classification by splitting the data into a test and a training set. For the feature selection and the training of the classifier we are using the training set only. Then, we use the test set to estimate the performance of the classifier, considering the selected features and the settings learned during the training phase. This procedure is repeated *m *- 1 times (*m *= 43). The Weka data mining software [43] has been used for classification.

## Results

In the following we describe the results and also compare our method to non descriptor-based alternative approaches.

### Univariate Analysis

**Figure 2:**
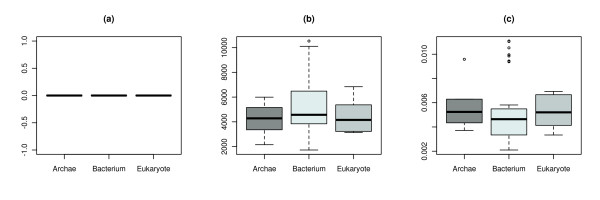
**Boxplots for basic network descriptors**. This figure shows the boxplots of (a) the global clustering coefficient, (b) the edge density, and (c) the average distance. These three basic structural features show no ability to discriminate between the three domains of life.

The three descriptors that show the least group-specific effect in terms of an analysis of variance (ANOVA) are shown in Figure 2. In our particular case, it relates to the edge density [44], the average distance [45], and the global clustering coefficient [46]. These measures capture basic topological properties. This is in accordance with the findings of Jeong et al.

ANOVA also identifies seven topological network descriptors that show a group-specific effect in at least one domain (*q*-value *<*0.1) as listed with their respective significance levels in Table 1. The choice of *q <*0.05 would have reduced the number of significant descriptors to two. We chose a higher significance level to motivate and stimulate discussion of this network based analysis approach.

**Table 1:** Top ranked features by ANOVA

Topological Network Descriptor	*q*-values
BERTZ complexity index (*C*)	0.013428
Radial centric info index (*I*_*C*,*R*_)	0.024471
Balaban *J *index (*J*)	0.059016
Dehmer-Entropy using *j*-spheres $\left(I_{f_{exp}^{V}}\right)$	0.059016
Dehmer-Entropy using path lengths $\left(I_{f_{\text{exp}}^{P}}^{\lambda }\right)$	0.059016
Dehmer-Entropy using vertex centrality $\left(I_{I_{lin}^{C}}^{\lambda }\right)$	0.065820
Vertex degree equality-based information index (*I_deg_*)	0.065820

This table shows the topological network descriptors that show a group-specific effect in at least one domain (*q*-value *<*0.1), identified by ANOVA. A description of of the different descriptors (features) can be found in the additional file.

**Figure 3:**
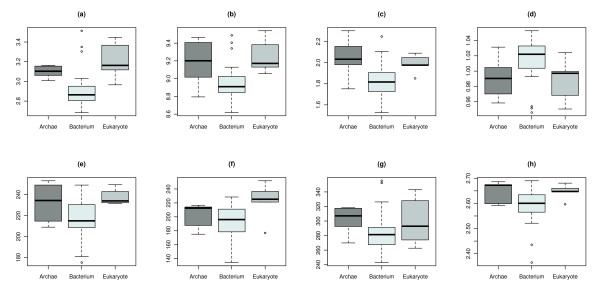
**Boxplots for the significant descriptors by ANOVA**. This figure shows the boxplots of eight significant topological network descriptors: (a) The Bonchev - Trinajstić index, (b) the Compactness, (c) the Radial centric information index, (d) the Balaban *J *index, (e) Dehmer entropy using *j*-spheres, (f) the Dehmer entropy using path lengths, (g) the Dehmer entropy using vertex centrality and (h) the vertex degree equality-based information index, as listed in Table 1.

This shows that five of the seven descriptors are from EBD. This strengthens our hypothesis that entropy-based descriptors perform better at classifying biological networks [12]. The values of these significant descriptors are illustrated as boxplots in Figure 3. One can see that one single descriptor is insufficient for discriminating between all three domains of life. To overcome this shortcoming we employ a multivariate approach as described in the Methods section.

### Feature Selection

Table 2 depicts the five top-ranked features according to mean score and standard deviation (sd) for the feature ranking methods ANOVA, IG, and RF. Lower ranked features are not shown as the IG converges to zero. LOOCV was applied to validate the feature ranking scores. The common set of selected features comprises the Bonchev-Trinajstić index *I_D _*[47], and the Compactness *C *[48]. Their boxplots are shown in Figure 3.

**Table 2:** Top ranked features

Feature ranking method			
**Rank**	**Anova** **(mean ± sd)**	**IG** **(mean ± sd)**	**RF** **(mean ± sd)**

1	*I_D _*(8.78 ±1.27)	*I_D _*(0.44 ± 0.05)	*I_D _*(0.18 ± 0.01)
2	*C *(7.29 ± 0.82)	*C *(0.08 ± 0.14)	*C *(0.14 ± 0.01)
3	*I_C,R _*(5.96 ± 0.68)	*I_C,R _*(0.05 ± 0.12)	*J *(0.11 ± 0.01)
4	*J *(4.12 ± 0.58)	$I_{f_{\text{exp}}^{P}}^{\lambda }$ (0.02 ± 0.08)	$I_{f_{lin}^{V}}$ (0.11 ± 0.01)
5	$I_{f_{\text{exp}}^{P}}^{\lambda }$ (4.04 ± 0.65)	$I_{I_{lin}^{C}}^{\lambda }$ (0.01 ± 0.04)	$I_{f_{exp}^{V}}$ (0.105 ± 0.01)

This table shows the five top ranked features using different feature selection methods. A description of the different descriptors (features) can be found in the additional file.

### Supervised Machine Learning

Performing a classification using the five best features for each of the two groups (EBD and NEBD) leads to a reasonable performance. The best results are achieved with the logistic regression. NEBD achieved a lower classification performance with a weighted *F*-score (WFS) of 74.1% and an accuracy (ACC) of 79.1% versus EBD with a WFS of 70.5% and an ACC of 72.1%.

Combining all the descriptors of the two groups, *k*-nn and logistic regression both gave the highest accuracy and *F*-Score using RF for feature selection. However, in contrast to *k*-nn, logistic regression is model-based, where the training data is compiled into a concise model that can be further interpreted [38,49]. Therefore, we focused on the results of the logistic regression. It achieved a WFS of 83.7% and an ACC of 83.9% as shown in Table 3. The corresponding confusion matrix is depicted in Table 4. It shows that the classification of the three domains of life using topological graph measures leads to a reasonable classification performance.

**Table 3:** Performance measures of different classifiers

	ANOVA			IG			RF		
Classifier	ACC %	MAE	WFS	ACC %	MAE	WFS	ACC %	MAE	WFS
*k*-nn	83.72	0.13	0.82	79.07	0.16	0.79	83.72	0.13	0.83
Naive Bayes	74.42	0.18	0.75	76.74	0.21	0.76	72.09	0.19	0.73
logistic regression	79.07	0.17	0.76	81.40	0.13	0.83	83.72	0.14	0.83

This table shows the Accuracy (ACC), mean absolute error (MAE), and weighted average *F*-score (WFS) for *k*-nn, Naive Bayes, and logistic regression, using leave one out cross-validation.

**Table 4:** Confusion matrix for *k*-nn and IG

True class	Archaea	classified as Bacteria	Eukaryotes
Archaea	2	4	0
Bacteria	3	29	1
Eukaryotes	0	0	5

This table shows the confusion matrix for the features selected by IG and the *k*-nn classifier.

In order to verify the discrimination ability of the classifier we compared its performance with the one derived from a data set where the class labels for the samples were randomly permuted. The average performance of this classifier leads to an WFS of 58.5% and an ACC of 58.5%. These results show that the performance reduces substantially compared to the original classifier for predicting the three domains of life.

### Comparison with Non Descriptor-Based Methods

To assess the classification ability of our approach we also compare it to non descriptor-based methods. As previously mentioned, we could not apply the generalized Zelinka distance using subgraph isomorphism [22,23] because of its infeasible computational complexity and the size of our networks. Therefore, we employ an information-theoretic approach using the degree distribution of each network and the Kullback-Leibler divergence [50]. Subsequently, we perform a supervised machine learning approach as described above. Using the Kullback-Leibler divergence we find no relevant features having a mean information gain *>*0.01. The best classification performance without feature selection is obtained using logistic regression (ACC = 53.48%, MAE = 0.31, WFS = 0.54).

**Figure 4:**
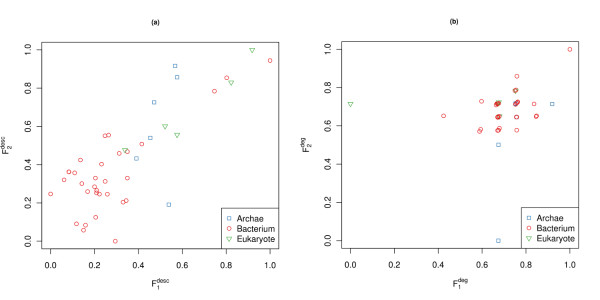
**The feature space for the distance-based approach**. To compare the two approaches, we visualize the feature space of the two top-ranked features by ANOVA. (a) represents feature space of the descriptor-based approach where $F_{i=1,2}^{desc}$. (b) represents feature space of the non distance-based approach where $F_{i=1,2.}^{deg}$. *^deg^*. To gain the features vectors in (b), we calculate the Kullback-Leibler divergence (KLD) based on the degree distribution between each network. This leads to a feature vector for each network containing the KLD for each of the other networks.

To visualize the difference between the descriptor-based method and the approach using the Kullback-Leibler divergence, we plot the feature space spanned by the two best features as reported by ANOVA. Figure 4(a) shows the descriptor-based approach where it is possible to identify three clusters. In Figure 4(b) the data presents itself as one single cluster.

## Discussion

The goal of our work was to discriminate between the three domains of life Archae, Bacterium, and Eukaryote, based on a set of 43 metabolic networks. Therefore, we utilized topological network descriptors. This was a non-trivial task because we had to identify descriptors that are able to capture domain-specific topological network characteristics meaningfully. We first employed a univariate approach to test the topological network descriptors for a domain-specific effect. In a second step we applied feature selection and supervised machine learning techniques.

The corresponding results indicated that despite the topological similarities shown in Jeong et al. [2], we were able to specify differences between the three domains of life. This demonstrates that the measures used can capture significant structural information. Since QuACN can only process undirected networks, we disregarded the information on the direction of the edges. However, we showed that the topology of the metabolic networks still contains enough information for discrimination between the three domains of life. These findings indicate that despite some existing topological similarities, the domains of life might have developed specific topological properties in their related metabolic networks. Based on these conclusions it might be worthwhile to investigate whether such specific structures and topological properties can also be found on other taxon levels.

The basic topological descriptors (global clustering coefficient, edge density, and average distance) showed no sufficient classification ability for this set of network data, when applying ANOVA. Thus, we employed two groups of more sophisticated descriptors (entropy-based and non entropy-based). We could demonstrate that different groups of topological network descriptors perform differently on this set of networks. The group of non entropy-based descriptors achieved the lowest results. This demonstrates that the non entropy-based descriptors have a lower classification ability than the entropy-based ones, for this set of metabolic networks.

**Figure 5:**
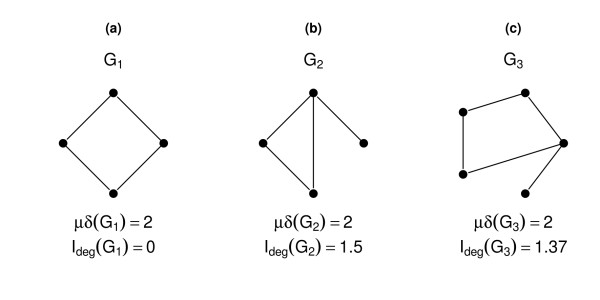
**Example for graph Sensitivity**. This figure shows three simple networks, their corresponding mean degree (*μδ*(*G_i_*)), and the entropy of the degree distribution (*I_deg_*(*G_i_*)).

This can be explained by the fact that entropy-based descriptors are often more sensitive in capturing structural differences than are classical network descriptors [8,47]. Consider the following simple example as illustrated in Figure 5. It shows three small, structurally different networks. However, the mean of the degree of these three networks produces the same result for each network, i.e., *μδ*(*G*_1_) = *μδ*(*G*_2_) = *μδ*(*G*_3_) = 2. However, comparing this to the entropy of the degree distribution [9] given by

(1) $$I_{deg}=-\overset{k}{\underset{i=1}{\sum }}\frac{\left|\delta_{i}\right|}{\left|\delta \right|}\log\frac{\left|\delta_{i}\right|}{\left|\delta \right|},$$

where |*δ_i_*| denotes the number of vertices with the same degree and *k *denotes the maximum degree, this results in different values for each network (*I_deg_*(*G*_1_) = 0, *I_deg_*(*G*_2_) = 1.5, *I_deg_*(*G*_3_) = 1.37). This small example demonstrates that slight changes of the network topology cannot be detected by the mean degree. On the other hand, the degree distribution is different for each network, and so is the entropy. Moreover, this demonstrates that this particular entropy-based measure is more sensitive to small changes in the topology of a network. But this does not mean that entropy-based measures do not have any degeneracy [8,27]. Generally, a measure is called degenerated if there exist two non-isomorphic graphs possessing the same value, see [51]. Note that the problem of evaluating the degree of degeneracy has been studied in, e.g., [8,27]. In particular, Dehmer et al. demonstrated that parametric entropy measures possess higher uniqueness than partition based descriptors [27]. Also, Konstantinova demonstrated that information indices based on distances show a high discrimination ability [52].

With respect to our results, this indicates that the information content, as captured by entropy-based measures, often allows a more meaningful discrimination than using other graph invariants. This fact finally leads to a better classification performance. This hypothesis was supported using one way ANOVA where we were able to identify seven descriptors having a significant group effect, and five of them (71.4%) were entropy-based. Additionally, combining the two groups and performing a feature selection showed that four of the five selected features were entropy-based. This feature selection showed that already five features were sufficient for identifying the three domains of life from the present data.

Moreover, in recent work we have demonstrated that it is necessary to combine descriptors from different groups in order to exploit their ability to capture different structural network features in order to increase their classification performance [53]. This demonstrates that combining different structural features can increase the ability to discriminate between different classes of networks. However, it is challenging to identify the structural features and the set of topological network descriptors that can capture these features.

Due to the imbalanced class distributions, a high overall classification performance is achieved. Consider a two class problem with classes A (*n_A _*= 10 instances) and B (*n_B _*= 90 instances). A naive classifier that labels all classes as B (majority class) will achieve an accuracy of about 90%. In our case, a classifier that would always classify a network as Bacteria would achieve an WFS of 63.5% and an ACC of 74.4%. In the context of this observation, we achieved a fairly respectable result for this imbalanced data set.

Moreover, we randomized data to show whether we capture something essential or simply overfit the data. Therefore, we permuted the class labels randomly and applied the same algorithm on the data. This led to a WFS of 58.5% and an ACC of 58.5%. These results show that the performance decreases substantially by randomizing the class labels and indicates that we capture essential structural information of the underlying metabolic networks.

Comparing our method with alternative approaches showed two major findings. Methods using subgraph isomorphism such as the generalized Zelinka distance [23] could not be computed for such large networks, because of their high computational costs. Moreover, when one has *n *networks, all networks have to be compared with each other, leading to $\frac{n\left(n-1\right)}{2}$ computationally intensive comparisons. By using our approach, it is not necessary to compare all networks with each other, as a set of topological descriptors is calculated once for each single network. Note that with an approach based on the Kullback-Leibler divergence using the degree distribution, only one descriptor has to be calculated. Although this is important, this approach fails to produce meaningful classification results.

Our results demonstrate that it is possible to classify networks into three different domains of life, using only the topological characteristics of their underlying metabolic networks. Note that this method can be easily adapted to other types of biological networks.

## Conclusions and Outlook

By transforming the data into undirected networks, part of the information gets lost. As the implementation of QuACN is only capable of handling undirected networks, this package will have to be adapted in future work.

The results of the classification achieved in this study have motivated us to perform a study with an extended number of networks in each domain of life. To obtain a more equally distributed set of networks among the three domains of life, we plan to integrate different databases to increase the number of networks for each domain. To deal with this problem, we will also investigate other groups of topological network descriptors.

To obtain a better biological interpretation of the results, we plan to analyse the topology of every single metabolic network in detail. Based on the present findings it might be possible to derive species-specific topological information that can be used to track evolutionary events and modifications. So far, there does not exist a study that investigates which biological characteristics are captured by topological network descriptors, but we plan to perform one in future work. This will help us to identify important metabolites for the biological interpretation of the data.

The starting hypothesis of this work was that applying complex network measures is a feasible way to classify between metabolic networks that represent the three domains of life. We verified this hypothesis by finding a set of measures that allows a sufficient classification. These results demonstrate the power of such complex network measures and their potential to tackle challenging questions in network biology [8].

## Competing interests

The authors declare that they have no competing interests.

## Authors' contributions

LAJM, KGK and NM performed the computations and LAJM, KGK, NM, AG and MD interpreted the results. MD initiated and supervised the study. LAJM, KGK, NM, AG and MD wrote the paper. All authors created the manuscript and approved the final version.

## Reviewers' comments

### Reviewer's report 1

#### Arcady Mushegian, Stowers Institute for Medical Research, Kansas City, Missouri, United States of America

In this manuscript, "metabolic networks" are studied, i.e., the graphs abstracted from tentative reconstructions of metabolic pathways in 43 species from the three domains of Life. The pathways and their graph representation were the same as used by Jeong et al. [1], and they go back to the WIT resource, produced by R. Overbeek in the 1990s. The authors of the current manuscript computed a large variety of topological descriptors of complex networks, many of which have been proposed only recently (some by the authors themselves), and applied various statistical machine learning techniques to find the descriptors or their combinations that best separate three domains of Life, i.e., Bacteria, Archaea and Eukarya. The main conclusion of the study can be found on p. 9: "These findings indicate that despite some existing topological similarities, the domains of life might have developed specific topological properties in their related metabolic networks. Based on these assumptions it might be worthwhile to investigate whether such specific structures and topological properties can also be found on other taxon levels."

I am not an expert in machine learning methods and trust the others the evaluate the methodology. (But see one technical remark at the end). In this review, I would like to focus on the biological sensibilities of the journal and its audience, and to invite the authors to explain better what is the goal of the whole exercise is. Their motivation is mentioned several times, but the statements of the problem are confusing. For example, on p. 2 we read: "The approach introduced in this paper determines structural features by utilizing topological network descriptors, in order to classify metabolic networks of 43 organisms", and yet the title says that the project attempted to classify the three domains of Life - these are not the same, which one was it?

If we go with the title, we find that the authors' understanding of classification and the one used by many biologist may not be the same. The authors seem to be more concerned in using their quantitative approach to achieve good separation of the objects with the known labels, whereas a biologist sees a classification as the knowledge base about relationships between organisms, and looks for a better way to classify a newly sequenced organism. The classifiers proposed here do not seem to be immediately useful for either purpose. Moreover, the "metabolic network" that the authors use is itself a result of many steps of computational analysis, starting from gene recognition, going on to similarity detection and metabolic reconstruction. Using this highly derived metabolic map to answer a basic question of whether this organism is from Bacterial, Archaeal or Eukaryal domain seems contrived: molecular properties of protein and RNA genes would have allowed one to assign the organism quite unequivocally at a much earlier step of the analysis, horizontal gene transfer notwithstanding in this case.

But perhaps this study should be taken on its own merits, to show that even a partial, incomplete as it was in the late 1990s, information about metabolic pathways in three domains of Life, was sufficient to detect informative topological differences between the networks in different domains. How interesting this observation might be? In my opinion, it depends on the nature of the signal that is detected by the authors' approach. Could it be that it simply registers the pathways that are not shared by the three domains of Life? Could it reflect the incompleteness of relatively conservative metabolic reconstruction in the (now superseded) WIT database, which reflects perhaps mostly the peculiarities of biochemistry in Archaea which were known and examined prior to 1990s? Or could it be that not enough of Archaeal diversity (or, for that matter, the diversity of all three domains of Life) was sampled in 2000?

If, on the other hand, the sophisticated measures that the authors use do indeed capture some not-biologically-intuitive property of a metabolic network that is specific to, say, bacteria, such as different patterns of local connectivity, or different patterns of centrality, etc., this would be worth knowing -though, in this case again, one wonders how all this would hold up with many more genomes and their (putative) metabolic networks available now than was known in 2000.

#### Author's response

The reviewer addresses a very important issue. There is often a terminological misunderstanding between experts from different areas. We want to make clear that we understand the term classification in a methodical way. More precisely, the term classification is based on using *supervised machine learning *and the goal is to use training data to predict future class membership of a sample or certain characteristics of the whole data set [54]. In our case, this means that we use the values of the different topological network descriptors of the underlying metabolic network of an organism to predict which domain of life the organism belongs to.

In this study, we want to investigate whether it is possible to capture domain-specific topological similarities by using different groups of topological network descriptors. We want to emphasize that we are mainly interested in the methodical aspect. Therefore, we showed that the methods are able to detect topological properties which predict domain membership with a reasonable classification performance. We agree with the reviewer that it would be quite unequivocal to assign the organism at a much earlier step of the analysis. But, note that the presented approach is based on information intrinsic in metabolic networks that is captured by domain-specific topological properties.

We fully agree with the reviewer that it would be worth knowing what biological or non-biological properties are captured by these sophisticated measures and if the presented domain-specific topological properties can be generalized for many more genomes. As explained in the section *Conclusion and Outlook*, the results of this study motivate us to perform a large scale study with more recent metabolic networks.

### Reviewer's report 2

#### Carlo Vittorio Cannistraci, Integrative Systems Biology Lab, King Abdullah University of Science and Technology, Saudi Arabia

##### Synopsis

Muller et al. propose a network-based approach for classification of the three domains of life (Archaea, Bacteria, Eukaryota). The dataset is composed of 43 metabolic networks (6 Archaea, 32 Bacteria, five Eukaryota). Each network is codified by 33 features which are topological network descriptors able to capture domain-specific complexity in a meaningful way. They claim to demonstrate with ANOVA that 8/33 topological network descriptors are group-specific and, in particular, the entropy-based descriptors (6/8) are the most effective. In the second phase of the study they perform feature selection and classification (leave-one-out cross-validation is applied). They claim to attain high level of classification (accuracy *>*80%) confirming the superiority of the entropy-based descriptors (4/5 selected features). In addition, poor classification using non descriptor-based network codification is demonstrated.

##### General review

The main idea of the article is very elegant and the investigation of the topological descriptors to capture complex network properties is intriguing as much as is the comparison between the entropy-based descriptors (related to information theory concepts) and the classical descriptors (related to measures of distances between nodes, node degree, etc). However, I believe that this study presents some serious methodological issues and that the results are misleading due to a fundamental error in designing the classification task. The consequence is that - in my opinion - the authors cannot claim to have classified the three domains of life, as they report in the title and in the rest of the article.

Major comments 1) The first and most relevant issue is the design of the classification task. In fact, it consists only of a training phase while the test phase is completely absent. This is a serious and fundamental issue as explained in Smialowski P. et al (Pitfalls of supervised feature selection - Bioinformatics 2009). A correct study should use a first partition of the original data as training set, which should be employed to perform the feature selection and to learn the classifiers' settings. The second partition of the original data should be used as test set to estimate the performance of the classifiers considering the selected features and the settings learned during the training phase. The classification result on the test set - evaluated by weighted *F*-score (WFS) and accuracy - should be finally used to claim the level of classification achieved on the three domains of life. The authors could assert that the dataset is imbalanced in the number of classes, and it represents an obstacle to derive training and test datasets. This is a problem that in general is solved by unsupervised classification, as suggested in Martella (Bioinformatics 2006) and Cannistraci et al. (Bioinformatics 2010). In order to prove that it is possible to unsupervisedly classify the three domains of life, the authors should apply different clustering algorithms both to the dataset composed of the 33 original features (first evaluation) and to the dataset composed of only the five selected features (second evaluation). The result of these comparisons should be used to claim a certain level of discrimination by unsupervised classification, and to investigate the difference in performance between the selected features and the original ones. The reason why one should apply different clustering algorithms and consider the best performance is to guarantee that the final evaluation is not penalized by the bad performance of a particular approach. Clustering approaches able to work both for spherical clusters (such as *k*-means; and Affinity propagation, Frey et al. Science 2007) and for irregular/elongated/non-spherical clusters (such as Minimum curvilinear Affinity propagation, Cannistraci et al. Bioinformatics 2010; and soft-constraint affinity propagation, Leone et al. Bioinformatics 2007) should be considered.

2) Multiple testing correction is applied and a q-value < 0.1 is used as threshold for significance to determine the descriptors with a group-specific effect in ANOVA: this identifies seven significant descriptors. In general, a common choice for significance is a q-value < 0.05 which would reduce the number of significant descriptors to 2. The authors do not motivate the choice of a q-value threshold so unexpectedly high (q-value < 0.1).

Minor comments 1) The name of the 33 topological descriptors should be indicated in the Methods section, considering each of the 4 types.

2) Methods section, feature selection paragraph: the authors state that feature selection methods can be classified into filters and wrappers. This is incorrect. Feature selection methods can be subdivided into at least three types: filter, wrapper and embedded (Yvan Saeys et al., A review of feature selection techniques in bioinformatics - Bioinformatics 2007).

3) In the paragraph "comparison to non-descriptor-based methods": it is not clear how many features were generated. Moreover, it is not clear what features are used to obtain Figure 4B.

#### Author's response

Answer to Major comments 1: The classification task includes leave-one-out cross-validation (CV) using *m *- 1 (*m *= the number of samples) partitions for training and the remaining part for testing the classifier. The whole procedure was repeated *m *times, and we calculated the mean *F*-score, absolute error, and accuracy. In our particular case *m *= 43.

We also validated the feature selection using leave-one-out CV and calculated mean ANOVA, information gain, and relief scores. To estimate the predictive ability of the descriptors and to compare supervised methods we used the set of five top ranked descriptors (according to mean scores) as input for classification. This procedure is called internal CV [42].

However, we fully agree with the reviewer that an external CV [42] is required to avoid any selection bias. Therefore, we now additionally performed external cross-validation using *m *- 1 partitions (*m *= 34) for feature selection and training, and used the remaining part for unbiased testing of the classifier. *k*-nn and logistic regression now yield both the highest accuracy and *F*-Score using RF for feature selection.

However, in contrast to *k*-nn, the logistic regression is model-based, where the training data is compiled into a concise model that can be subsequently interpreted [38,49]. Therefore, we focused on the results of the logistic regression and changed the related parts of the manuscript.

We also agree with the reviewer that there are unsupervised methods that can handle imbalanced datasets. But our aim is to use supervised machine learning, as the class labels are known. Motivated by the comments of Reviewer 3, we performed the classification task with randomized class labels to comparably assess the performance. We added these results to the subsection, *Supervised Machine Learning *in the section *Results*. This analysis shows that the performance drops significantly by randomizing the class labels, and that our method does detect class information essentially.

Answer to Major comments 2: We fully agree with the reviewer that the significance level of 0.1 for the *q*-value is high. However, a closer look at Table 1 reveals that a significance level of 0.066 would have led to the same number, seven, of descriptors. We decided to accept a higher threshold, and list all seven descriptors with respective significance levels as we felt that this threshold is more stimulating for the subsequent analysis and discussion of this metabolic network based classification approach. We added the explanation of our choice to the manuscript.

Answer to Minor comments 1: We added an additional file 1 with tables containing the descriptors used. Answer to Minor comments 2: The authors thank the reviewer for this comment. The enumeration previously only included the two basic feature selection types described by Baumgartner et al. [55] and has now been extended to embedded and ensemble-based methods.

Answer to Minor comments 3: As described in the section "Comparison to Non-Descriptor-Based Methods", we calculated the Kullback-Leibler divergence (KLD) based on the degree distribution between each network. In particular, this leads to a feature vector for each network containing the KLD for each of the other networks. These features were used to plot Figure 4(b). To make that clear we added a more detailed description to the text of Figure 4.

#### Reviewers comments about authors's response

The Authors did not provide a satisfactory answer to my major comments. In particular, they proposed an external CV procedure, which is different from what I asked, and far away from providing an answer to the concerns that I raised in the major comment 1 of my first review.

#### Authors comment

We thank the reviewer for his comments and appreciate his suggestions. However, we want to clarify that we performed the feature selection and the classification as suggested by the reviewer. Therefore, we re-performed the classification by splitting the data into a test and a training set and used cross-validation. In particular, we performed the feature selection and the training of the classifier using cross-validation only by using the training set. Then we only used the test set to estimate the performance of the classifier, considering the selected features and the settings learned during the training phase. This procedure has been repeated *m *- 1 times (*m *= 43) and note, that this is often refereed to as external cross-validation since the evaluation of the classifier is performed externally. See [41,42], as suggested by the reviewer. In particular, Smialowski et al. [41] state that any model building method integrated with feature selection must be *externally *evaluated and Abroise and McLachlan [42] declare that the same feature selection method must be implemented in each stage of an (*external*) cross-validation. To remove ambiguity, we now explicitly describe the used procedure in the section "Material and Methods" instead of simply calling it *external *CV.

Unsupervised techniques can be fruitful to handle imbalanced data sets [56,57], but we have not dealt with this problem as we put the emphasis on *graph classification *by using supervised machine learning only. But the usage of unsupervised techniques such as clustering [56] might be feasible for exploring the networks and their common structural features for several other research questions in the context of distinguishing between the three domains of life.

We are grateful for your comments, as they have improved the text and helped to understand an interdisciplinary problem more properly.

## Reviewer's report 3

### Christoph Adami, Keck Graduate Institute, Claremont, California, United States of America

In this contribution, the authors attempt to use the structural properties of metabolic networks to identify what domain of life they belong to, using a dataset of 43 previously published networks and a set of topological descriptors of the network, using 33 "descriptors" or features. The authors show that using feature selection as well as machine learning methods, they can classify the networks with an accuracy of over 88%, as compared to the 63.5% performance of always picking bacteria, the most abundant domain in the data set.

There are a number of points that require clarification.

Abstract: You discuss "two groups" of descriptors but do not mention what these two groups are. This is confusing the reader.

Materials & Methods: While I understand that it is not possible to describe each of the descriptors in the manuscript, there should at least be a Table of them, with perhaps a single line that summarizes the nature of the descriptor, as well as a reference. Without it, I find myself constantly looking for what abbreviations such as "ID, C, ICR, J, IfpexpDist" etc mean. In fact, I'm sure some of them are never defined in the text. Then, what good does it do to give me the rank of a feature (as in Tables 1) if I have no idea what these feature are?

You mention the software packet you use (QuACN) for the first time in the "Data" subsection, without introducing it, describing it, or telling the reader what the acronym stands for. In fact, the sentence "As the implementation of QuACN is only capable of ...." sounds as if you introduced it before, but you did not. "Leave-one-out cross-validation" is technically the incorrect term for what you are doing: it is really m-fold cross-validation, because you divide the dataset into m groups. In "Leave-one-out cross-validation" you would divide the data set into 43 groups, and cross-validate by leaving one out, 43 times. Unless *m *= 43, then they are the same. And you don't mention what m you use. You form two group of descriptors, but you do not give us a list of which descriptors are deemed to be entropy-based and which aren't. You can give that information in the Table that summarizes the 33 features.

Results: The results of the univariate analysis are not presented well. Table 1 lists 8 descriptors (most of them obscure as we have not been given even the most cursory description of them) and that they show a group effect "in at least one" domain. Would it be so difficult to give the F-statistic for each domain? Also, it is curious that the generally accepted practice of using a 5% significance level has been replaced by a 10% level without any discussion. Could it be that it was done so that the authors' parametric entropy makes the cut???

You claim that NBD measure do not perform as well as EBD measures in classifying the domains (the paragraph that starts with "The basic topological descriptors...."), but it is not at all clear whether the performance score differences you give are significant, because I suspect that the number of features in each set is not equally balanced. For the life of me, I cannot find these numbers (how many descriptors of each kind) in the manuscript. You really need that Table. Whether a weighted *F*-score of 83.2% vs. 86.2% is significant is really not clear at all.

It is also surprising that you do not present the results of a principal component regression analysis of the features to determine how much each feature explains the variation in the three domains.

Moreover, it is not clear whether your method will be able to use the features to successfully predict the domains using data that was not used for training. It is well known that machine learning techniques can explain even random data to some degree. One way to test this is to scramble the data so that networks are given random domains (but leaving the relative numbers the same. that is 32 bacterial networks, six arch, and five eukaryotic networks). After this, apply the learning algorithm exactly as before. If you can recover this (obviously bogus) classification using your machine learning algorithm, then all you did was fit the data. But if the prediction accuracy falls significantly, then you are indeed capturing something essential using the structure-based descriptors.

Finally, English language is strongly deficient in parts. Please ask a native speaker to go over the manuscript.

#### Author's response

We thank the reviewer for his comments, and agree that we did not introduce the two groups in the abstract but emphasize that they have been introduced in detail in the section *Material and Methods*. Hence, we think it would be more confusing to the reader if we introduce the two groups within the limited space of the abstract, without a detailed description.

We fully agree with the reviewer that a table with the used descriptors is necessary to interpret our results. Therefore, we added a file that lists the different used topological descriptors.

As the R-package QuACN was not introduced correctly in the *Data *subsection, we now introduce the package in the section *Background*.

The reviewer is right that we did not mention the value for *m*. However, *m *= 43, so *Leave-one-out cross-validation *is technically the correct term for the applied CV. We now specify the value *m *in the section *Methods*.

The choice of 0.1 for the significance level of the *q*-value is already explained in the response to the comments of Reviewer 2. However, we only performed the univariate analysis to motivate the classification. If there were not more descriptors within this significance level of 0.1, further analysis would not have been promising. We added the explanation of our choice to the manuscript.

The reviewer is right that the number of descriptors is not the same in the two groups of descriptors and that it is not clear whether the WFS is significant by comparing the two groups (NEBD vs. EBD) without knowing the amount of descriptors in each group. As mentioned before, we added an additional file containing a description of the groups of descriptors. Moreover, motivated by the comments of Reviewer 2 we performed the whole analysis again using external feature selection. This leads to new unbiased WFSs that can be compared because the classification was done by using the best five features within each group. Note that our aim is to classify our data using (*supervised machine learning*). So we did not perform a PCR analysis because that would not shed light on the classification problem. In our understanding, the dependent variable has to be numeric for a regression analysis and in our case it is discrete (Archaea, Bacteria, Eukaryote). Moreover, as we understand a PCR, it would show how much each feature explains the variation in each principal component and not in the three domains. However, performing a PCA did not cluster the networks in a meaningful way.

We thank the reviewer for the recommendation to perform the analysis with randomized data to show whether we capture something essential or simply overfit the data. Therefore, we permuted the class labels randomly, as recommended by the reviewer, and applied the same algorithm on the data. This led to a WFS of 58.5% and an ACC of 58.5%. These results show that the performance decreases substantially by randomizing the class labels and indicates that we capture essential structural information of the underlying metabolic networks. We added these results to the manuscript.

## Supplementary Material

Additional file 1 **Tables of Network Descriptors**. This file contains an overview of the descriptors used. Click here for file
