# Supplementary material for: A network-based approach to classify the three domains of life
Source: Biol Direct. 2011 Oct 13;6:53. doi: 10.1186/1745-6150-6-53 (PMC3226542; doi:10.1186/1745-6150-6-53)
Supplement: Additional file 1 — Tables of Network Descriptors. This file contains an overview of the descriptors used. [file 1745-6150-6-53-S1.PDF]

Additional file 1 - Tables of Network Descriptors

This additional file gives a brief overview about the used topological network descriptors including the reference to the corresponding publication.

Descriptors based on distances

Name	Symbol	Ref.
Wiener index	$W(G)$	[17]
Hararay index	$H(G)$	[3]
Balaban J index	$J(G)$	[1]
Compactness	$C(G)$	[10]
Mean Distance Deviation	$\delta\mu(G)$	[14]
Hyper-distance-path index	$D_P(G)$	[15]

Descriptors based on other invariants

Name	Symbol	Ref.
Index of total adjacency	$A(G)$	[6]
Zagreb group indices 1	$Z_1(G)$	[9]
Zagreb group indices 2	$Z_2(G)$	[9]
Randić index	$R(G)$	[11]
The complexity index B	$B(G)$	[6]
Normalized edge complexity	$E_N(G)$	[6]

Classical entropy based descriptors

Name	Symbol	Ref.
Topological information content	$I_{orb}^V(G)$	[12, 16]
Bonchev-Trinajstić index 1	$I_D(G)$	[7]
Bonchev-Trinajstić index 2	$I_D^W(G)$	[7]
BERTZ complexity index	$C(G)$	[4]
Radial centric info index	$I_{C,R}(G)$	[5]
Vertex degree equality-based ii.	$I_{deg}(G)$	[5]
Balaban-like information index U	$U(G)$	[2]
Balaban-like information index X	$X(G)$	[2]
Graph vertex complexity index	$I_V(G)$	[13]

Dehmer entropy with information functionals using

Name	Symbol	Ref.
the j-spheres	$I_{f^V}(G)$	[8]
path lengths	$I_{f^P}(G)$	[8]
vertex centrality	$I_{f^C}(G)$	[8]

Each descriptor of this group has several parameters, for details see [8]. Each functional was used two calculate two descriptors i) the entropy (I_f) and ii) the *distance* of the entropy from maximum entropy (I_f^λ) [8]. Moreover, we used 2 different settings for the weighting parameter c_i (lin, exp) for each descriptor; thus, leading to 12 different descriptors for this class.

References

[1] A. T. Balaban. Highly Discriminating Distance-Based Topological Index. *Chem.Phys.Lett.*, 89:399–404, 1982.

[2] A. T. Balaban and T. S. Balaban. New Vertex Invariants and Topological Indices of Chemical Graphs Based on Information on Distances. *J. Math. Chem.*, 8:383–397, 1991.

[3] A. T. Balaban and O. Ivanciuc. Historical Development of Topological Indices. In J. Devillers and A. T. Balaban, editors, *Topological Indices and Related Descriptors in QSAR and QSPAR*, pages 21–57. Gordon and Breach Science Publishers, 1999. Amsterdam, The Netherlands.

[4] S. H. Bertz. The First General Index of Molecular Complexity. *Journal of the American Chemical Society*, 103:3241–3243, 1981.

[5] D. Bonchev. *Information Theoretic Indices for Characterization of Chemical Structures.* Research Studies Press, Chichester, 1983.

[6] D. Bonchev and D. H. Rouvray. *Complexity in Chemistry, Biology, and Ecology.* Mathematical and Computational Chemistry. Springer, 2005. New York, NY, USA.

[7] D. Bonchev and N. Trinajstić. Information Theory, Distance Matrix and Molecular Branching. *J. Chem. Phys.*, 67:4517–4533, 1977.

[8] Matthias Dehmer. Information Processing in Complex Networks: Graph Entropy and Information Functionals. *Applied Mathematics and Computation*, 201:82–94, 2008.

[9] M. V. Diudea, I. Gutman, and L. Jäntschi. *Molecular Topology.* Nova Publishing, 2001. New York, NY, USA.

[10] J. K. Doyle and J. E. Garver. Mean Distance in a Graph. *Discrete Mathematics*, 17:147–154, 1977.

[11] X. Li and I. Gutman. *Mathematical Aspects of Randić-Type Molecular Structure Descriptors.* Mathematical Chemistry Monographs. University of Kragujevac and Faculty of Science Kragujevac, 2006.

[12] N. Rashevsky. Life, Information Theory, and Topology. *Bull. Math. Biophys.*, 17:229–235, 1955.

[13] C. Raychaudhury, S. K. Ray, J. J. Ghosh, A. B. Roy, and S. C. Basak. Discrimination of Isomeric Structures Using Information Theoretic Topological Indices. *Journal of Computational Chemistry*, 5:581–588, 1984.

[14] H. P. Schultz, E. B. Schultz, and T. P. Schultz. Topological organic chemistry. 4. Graph theory, matrix permanents, and topological indices of alkanes. *Journal of Chemical Information and Computer Sciences*, 32(1):69–72, 1992.

[15] R. Todeschini, V. Consonni, and R. Mannhold. *Handbook of Molecular Descriptors.* Wiley-VCH, 2002. Weinheim, Germany.

[16] E. Trucco. A Note on the Information Content of Graphs. *Bulletin of Mathemtical Biology*, 18 (2):129–135, 1956.

[17] Harry Wiener. Structural Determination of Paraffin Boiling Points. *Journal of the American Chemical Society*, 69(1):17–20, January 1947.
